# Intergenerational caregiving on mental health of middle-aged and older adults in China: empirical insights

**DOI:** 10.3389/fpubh.2023.1224062

**Published:** 2023-07-06

**Authors:** Xueling Yan, Wenjing Wu, Xiaoqin Chen, Guangming Xu, Shule Yu, Shen Li

**Affiliations:** ^1^Economics School, Sichuan University, Chengdu, Sichuan, China; ^2^Department of Psychiatry, Qingdao Mental Health Center, Qingdao, Shandong, China; ^3^Tianjin Anding Hospital, Institute of Mental Health, Mental Health Center of Tianjin Medical University, Tianjin, China; ^4^Economics School, Fudan University, Shanghai, China

**Keywords:** intergenerational caregiving, mental health, middle-aged and older adults, financial support, spiritual support

## Abstract

**Introduction:**

The impact of intergenerational caregiving on the mental health of providers remains a controversial topic, especially in countries like China where it is prevalent. Given the country’s aging population and recent liberalization of the two-child policy, understanding the effects of intergenerational caregiving on the mental health of middle-aged and older adult(s) individuals is crucial. This study aimed to explore the impact of intergenerational caregiving on mental health among middle-aged and older adult(s) individuals.

**Methods:**

We analyzed data from the China Health and Aging Tracking Survey (CHARLS) 2013, consisting of 6602 participants finally. Personal information, family structure, financial support, health status, and physical measurements were selected for analysis. Correlation and regression analyses were used for relationships among variables controlling for potential confounding variables. Mental health status was evaluated using the depression self-rating scale.

**Results:**

There is a significant positive effect of intergenerational care on the mental health of middle-aged and older adult(s) people. Additionally, we re-profiled intergenerational care variables by considering the number and length of intergenerational caregivers, and found that the effects remained significant. Furthermore, the effects of intergenerational care vary across subgroups based on gender, age, nature of usual residence, marital status, and physical health status. Finally, we identified two mechanisms through which intergenerational caregiving positively affects mental health: intergenerational financial support and intergenerational spiritual support.

**Discussion:**

These findings have important implications for policymakers, healthcare professionals, and family members in promoting the mental health of middle-aged and older adult(s) individuals in China.

## 1. Introduction

As the global population continues to age, the worldwide public health concern of cognitive decline associated with aging (1) has gained significant attention. This decline not only has detrimental effects on physical and mental health, but also places increased economic, physical, and psychological burdens on those providing care (2). Additionally, it leads to escalating costs for families, communities, and governments (3). This condition is projected to be most prevalent in low-and middle-income countries (LMICs) (4). Within this context, China, as a populous LMIC and home to the largest population in the world (5), grapples with a substantial health challenge in relation to cognitive impairment among its older adults population.

Meanwhile, China’s population age has experienced a rapid aging trend in the 21st century, with a decline in fertility rate and an increase in average life expectancy (6). By the end of 2016, individuals aged 60 years and above and 65 years and above accounted for 16.7 and 10.8% of the total population, respectively, with a large scale and fast growth rate of population aging (7, 8). In response to this phenomenon, the government in China introduced new concepts to address population aging, such as developing policies and social environments that promote filial piety, respect for the older adults, and healthy aging, emphasizing the importance of physical and mental health of the older adults.

Moreover, with the implementation of China’s “two-child” policy in 2016, the future trend of fertility level and population development has become a significant concern from all walks of life. Previous studies indicate that the willingness of women of childbearing age have a second child increases significantly if both parents can provide care for the child. With China’s public childcare service system and market not yet fully established, it is both traditional and practical for older adults to assist in caring for their grandchildren, particularly infants and toddlers under the age of three (9–14).

According to the “2015 Family Development Report, “based on a survey conducted by the China National Aging Center in 2014, the proportion of children aged 0–2 who are primarily taken care of by their grandparents reaches as high as 60–70% nationwide. Among them, 30% of children are solely under the care of their grandparents. Even after the age of 3 when children start attending kindergarten, approximately 40% of them are directly nurtured by their grandparents. As nearly half of the older adults in China provides intergenerational care for their children, it is essential to investigate the impact of intergenerational caregiving. The current literature on intergenerational caregiving primarily focuses on three aspects: its impact on children, such as labor supply (9, 15, 16); its impact on grandchildren, such as infant and child health (17); its impact on grandparents, such as their employment and retirement (14, 18, 19). For middle-aged and older adults who provide intergenerational care, the impact of caring for grandchildren is of greater concern, not only in terms of their ability to provide care, but also in terms of whether the intergenerational care system can be further expanded. With the implementation of China’s comprehensive “two-child” policy, the scale of intergenerational care is likely to increase, and this may place additional pressure on grandchildren.

Intergenerational care has been a popular topic of research in recent years. Most existing literature has focused on the effects of intergenerational care on the explicit physical health of middle-aged and older adults, such as daily activity ability, and self-rated health (20–22). In terms of the effects of intergenerational care on the mental health of middle-aged and older adults, existing studies using data from different samples have not reached a consistent conclusion. Compared to physical health, mental health issues resulting from intergenerational caregiving are often overlooked. Providing intergenerational care requires good and dominant physical health, and it is often those grandchildren who are physically healthy that are observed when middle-aged and older adults decide to provide care. However, mental health is implicit, latent, and not easily detected, but its impact on people is particularly important. Therefore, this study focuses on the impact of providing intergenerational care on the mental health of middle-aged and older adults people in the context of population aging and the comprehensive “two-child” policy. We also explore the possible mechanisms behind this effect, providing a better theoretical basis for future policy formulation.

The remainder of the paper is organized as follows: Part II provides a more detailed literature review on intergenerational care; Part III describes the data variables and empirical model; Part IV presents the analysis of the empirical results; Part V discusses the potential mechanisms behind the impact of intergenerational care on mental health; and the conclusion summarizes the main findings and provides suggestions for future research.

## 2. Literature review

### 2.1. Overview of intergenerational care theory

#### 2.1.1. Motivation theory of intergenerational caregiving

Intergenerational caregiving is a prevalent phenomenon in many cultures and has been subject to various theoretical explanations. Three main explanatory theorical frameworks have been proposed to account for the motivation behind intergenerational caregiving: kin selection, sociocultural norms, and intergenerational exchange model (23). According to kin selection theory, grandparents provide care for their grandchildren to increase their own reproductive success by ensuring the survival and well-being of their own offspring (the parents of the grandchildren). Therefore, maternal grandparents are assumed to provide more care for their grandchildren than paternal grandparents due to the greater need for assistance in child-rearing that mothers typically experience (24).

Sociocultural norms theory proposes that caregiving differences between paternal grandparents and maternal grandparents can be attributed to the socio-culture context in which they operate. Paternal grandparents are reported to be more interested in their grandchildren, particularly males, than maternal grandparents, which is attributed to the patrilineal family system prevalent in many societies (25).

The intergenerational exchange model examines intergenerational caregiving from both altruism and “rational” perspectives. Altruistic individual, such as the head of the family, control family resources and prioritize the welfare of all family members above their own interests. Family resources are allocated to achieve the Pareto optimal allocation of resources, which reflects an altruistic motivation (26). The “rational” grandparent perspective, on the other hand, involves a rational estimation of the costs and benefits of investing time and resources (both emotional and financial) in their grandchildren (27). Grandparents provide intergenerational care to strengthen their relationship with their children (the parents of grandchildren) and to enhance the possibility of reciprocity with their children (23).

In summary, the motivations behind intergenerational caregiving are complex and multifaceted, with various theoretical frameworks proposed to account for this phenomenon. These frameworks provide important insights into the factors that influence the caregiving behavior of grandparents and can inform interventions aimed at supporting intergenerational caregiving relationships.

#### 2.1.2. Theory of the consequences of intergenerational caregiving

Intergenerational caregiving can have both positive and negative consequences on the physical and mental health of grandparents. Three main theories explain the consequences of intergenerational caregiving effects on grandparents: stressor-stress theory, role theory, and family stress theory (23).

Stressor-stress theory proposes that stressors experienced by grandparents in intergenerational care can lead to primary stressors such as accepting the caregiving role, and secondary stressors resulting from difficulties in managing and coping with caregiving responsibilities. When grandparents assume caregiving roles at inopportune times, stress may arise. The resulting stress from intergenerational parenting can negatively impact the psychological well-being of grandparents, including feelings of stress, frustration, depression, and reduced well-being (28).

According to role theory, there are two main perspectives: role burden theory and role optimization theory. The former suggests that individuals who take on multiple social roles face a range of demands or conflicts (23). When grandparents assume the responsibility of caring for their grandchildren, they may experience role conflict, leading to negative emotions such as frustration, depression, and adverse effects on their physical and psychological health. Conversely, the latter proposes that individuals can gain social integration and satisfaction from diverse social roles, which can offset the risks associated with social role burden, and enhance individual well-being. Therefore, grandparents who provide intergenerational care may increase social role diversity, which could potentially improve their overall health.

Family stress theory is a framework used to explain the differential effects of caregiving responsibilities on grandparents (23). This theory suggests that grandparents may experience more positive emotions when the family unit possesses personal and social resources, or when grandparents hold a positive attitude toward their caregiving role. On the other hand, a lack of formal or informal social support, or blurred role boundaries, may lead to negative emotional feelings. Thus, the impact of intergenerational caregiving on grandparents’ health status is dependent on their attitudes toward the caregiving experience. A positive attitude is likely to result in positive health outcomes, whereas a negative attitude is likely to lead to negative health outcomes.

In conclusion, the consequences of intergenerational caregiving on grandparents’ health are complex and dependent on various factors. Understanding these factors and their implications is essential for identifying potential risks and protective factors to improve the well-being of grandparents in caregiving roles.

### 2.2. Review of empirical studies on the impact of intergenerational caregiving

In terms of the prevalence and intensity of intergenerational caregiving, older adults in China have been found to provide more intergenerational care compared to other countries. The 2014 national survey of the China Social Tracking Survey of the older adults revealed that 73.29% of the elder adults in China provide intergenerational care for their grandchildren, with nearly 20% providing care at least once a week. On average, each older adults person provides care for 0.52 grandchildren. In contrast, a survey by Vega (29) of native-born seniors in the United States found that only 8.03% of seniors provide intergenerational care for their grandchildren, with only 2.36% providing care for more than 4 h per week. This difference may be due to traditional attitudes toward family and caregiving in China. Given the high prevalence of intergenerational caregiving in China, it is crucial to investigate the health impact of older adults who provide care.

However, the impact of intergenerational care on the health of older adults remains inconclusive due to divergent research perspectives, methodologies and samples. Some researchers argue that intergenerational caregiving has positive impacts on the health of the older adults. Various studies have indicated that engaging in social connections and productive activities can have positive effects on both physical and mental health for the older adults (30). For instance, participating in grandparent caregiving, which is prevalent in China due to its unique social and cultural context, has been associated with improved cognitive function and better overall well-being (31). Notably, the role of grandparent caregiving is often assumed by grandmothers, as they are traditionally regarded as primary caregivers (32), particularly in cases of intensive grandchild care (33). Some studies have suggested that caring for grandchildren may help maintain cognitive abilities in the older adults, as it provides positive experiences and requires cognitive resources, potentially delaying cognitive decline (34). Additionally, engaging in activities with grandchildren, encouraging physical exercise, and fostering social participation may contribute to enhanced cognition and a more vibrant lifestyle (35). Sun (36) also found that caring for grandchildren had better psychological health outcomes for rural older adults people, attributed to the traditional Chinese “community reasoning,” where adult children are rewarded for providing care to the older adults, making care for their grandchildren psychologically beneficial to the older adults. Coall and Hertwig (20) also found that high-intensity caregiving was more protective of grandparents’ cognitive function than low-intensity caregiving, supporting the “use it to get ahead” theory of aging activities. Jing et al. (21) studied national survey data of the China Social Tracking Survey of the older adults in 2014 and pointed out that intergenerational care provision was negatively associated with loneliness among the older adults. They posit that the positive impact of intergenerational care on mental health may be related to the family concept and the realization of self-efficacy among the older adults in China.

On the other hand, other researchers suggest that providing intergenerational care can have a negative impact on the health of older adults. For instance, it has been found that the responsibilities and demands associated with caregiving, such as increased stress levels, reduced personal time, and limited social engagement, may adversely affect cognitive function and mental health in some cases (37). Furthermore, while research has explored factors like caregiving frequency (37), intensity (38), number of grandchildren (39), and cohabitation (40). Lee et al. (41) found that older adults who provided care for their grandchildren had a higher number of chronic diseases and poorer physical condition than those who did not. Jing et al. (21) studied that the intensity of intergenerational caregiving and found that it can have a negative impact on the self-rated health of older adults. They concluded that providing care for grandchildren can be physically exhausting and increase the burden on older adults, leading to a lack of rest and the development of other diseases. Using the 2013 China Health and Aging Data Tracking Survey, Lo and Liu (22) found that providing intergenerational care was negatively associated with three aspects of older adults: impairment in daily activities, self-rated health status, and mental health status. The author attributed these negative effects to four reasons: tedious and repetitive caregiving, postponement of medical needs by grandparents, impact on grandparents’ social activities, and high caregiving stress.

It is imperative to consider the heterogeneity of caregiving families and individuals when investigating of the impact of intergenerational care on the health of older adults. The backgrounds of the older adults providing care, grandchildren receiving care, and the adult children entrusted with care vary considerably, making precise policy recommendations challenging. Sun (36) found that grandchild care provided by sons was beneficial to the mental health of older adults in rural China due to the influence of the traditional “son preference.” However, grandchild care provided by daughters was only beneficial to the mental health of male older adults, and had no effect on female older adults. The findings by Coall and Hertwig (20) showed that cognitive decline among grandparents caring for grandchildren was influenced by the grandparents’ age and gender, with a significant protective effect of intensive care on grandfather’s cognitive decline, while grandmother’s cognitive decline was exacerbated, and this gender difference widened with age.

In previous studies, there has been inconsistency in the direction of intergenerational caregiving on the mental health of middle-aged and older adults, with both positive and negative findings. It is crucial to note that the relationship between grandparent caregiving and cognition is a complex, multidimensional concept that requires further investigation. Additionally, it is worth mentioning that most existing research has focused on Western countries, leaving China and other LMICs understudied in this context. Moreover, there has been a dearth of studies on the impact of intergenerational care on the mental health of older adults in China, and most of the samples selected are local samples, which limits the generalization of findings. Finally, previous studies have not explored the mechanisms behind the effects of intergenerational care, which has implications for the scientific rigor of the research. To address these gaps, future research should explore the heterogeneity of caregiving families and individuals, consider larger and more representative samples, and investigate the mechanisms through which intergenerational care influences the mental health of older adults.

## 3. Data and methodology

### 3.1. Data

The present study utilized data from the China Health and Retirement Longitudinal Study (CHARLS 2013), which were collected and published by the National Development Research Institute of Peking University. Using a multistage probability-proportional-to-size (PPS) sampling technique (42), CHARLS surveyed with 28 provinces, 450 villages or communities in 150 districts or counties covered, and 23,000 individuals aged 45 years and older in 12,400 households were investigated in 2015 (43). The survey provides a large sample size, wide coverage, and good representativeness. The CHARLS 2013 dataset covers various demographic characteristics, family structure characteristics, living habits characteristics, economic and cultural characteristics, making it an ideal resource for this study (44).

Table 1 shows descriptive statistics based on data from the CHARLS 2013, indicating that nearly half of the older adults in China provide intergenerational care for their children. The dataset comprises high-quality microdata from 18,594 middle-aged and older households and individuals aged 45 and older (excluding data from 152 respondents aged under 45). The questionnaire administered during the survey included basic personal information, family structure and financial support, health status, physical measurements, health care utilization and health insurance, work, retirement and pensions, income, consumption, assets, and basic community information.

**Table 1:** Whether older adults help their children with childcare.

Gender	Yes	No	Total
Female	2,029 (26.75%)	1,912 (25.21%)	3,941 (51.96%)
Male	1,753 (23.11%)	1,890 (24.92%)	3,643 (48.04%)
Total	3,782 (49.87%)	3,802 (50.13%)	7,584 (100%)

Data source: CHARLS database (2013).

In this study, relevant questions related to basic personal information, family structure and economic support were selected based on the specific research needs. We selected 6,602 participants according to the following criteria: (1) aged 45 and older; (2) provided information on both grandparent caregiving and cognitive function. We also excluded the missing values of the main variables of the study.

### 3.2. Identification strategy

The objective of this study is to investigate the impact of intergenerational caregiving on the mental health of middle-aged and older adults parents, examine whether the impact is heterogeneous, and identify the possible mechanisms of impact. To achieve this aim, we designed the following model:


(1) $$\begin{array}{l} \mathrm{CESDscore}=\alpha +\beta \mathrm{babysit}+\gamma_{1}\mathrm{gender}+\gamma_{2}\mathrm{age}+\\ \gamma_{3}\mathrm{edu}+\gamma_{4}\mathrm{marriage}+\gamma_{5}\mathrm{residence}+\\ \gamma_{6}\mathrm{lgPCE}+\gamma_{7}\mathrm{fall}+\gamma_{8}\mathrm{ADL}+\varepsilon \end{array}$$

The CESDscore is the depression scale score of older individuals who provide intergenerational care, and it is used as the dependent variable to measure the mental health of middle-aged and older adults. To measure the mental health of middle-aged and older individuals, we selected 10 questions from the CHARLS Cognition and Depression Questionnaire. Following Lei et al. (45) we assigned scores of 0–3 to the options, with the highest level of depression assigned a value of 3, and the lowest level of depression assigned a value of 0. We summed the scores of these 10 questions to obtain each individual’s depression score, where a higher score represents lower psychological well-being (46). confirmed the high reliability of this scale in measuring the mental health of individuals.

The core explanatory variable in this paper is babys is, which represents whether or not intergenerational care is provided. We focused on its effect on the mental health of middle-aged and older adults. The question on the CHARLS questionnaire regarding intergenerational care is: In the past year, did you or your spouse spend time caring for your grandchildren or grandchildren? If yes was selected, babysit was equal to 1, otherwise, it was equal to 0.

To control for potential confounding factors, we selected several control variables. The first aspect includes demographic characteristics variables of the older adults, including gender( gender ), age ( age ), educational level ( edu ), marital status ( marriage ), and permanent residence nature ( residence ). The second aspect includes health status variables of the older adults, including fall - whether they fell in the last 2 years, and ADL - their ability to perform daily living. The third aspect includes the older adults economic status variables, specifically lgPCE - household *per capita* expenditure logarithmically, measured as annual household expenditure divided by the number of people eating at home, to avoid biasing the measurement by having family members working outside the home. The definition and construction of all control variables are shown in Table 2.

**Table 2:** Variables and indicators.

Variable	Definition	Description
CESDscore	Depression self-rating scale score	The higher the score, the worse the mental health status
Gender	Gender	Female = 0, male = 1
Age	Age	45–55 years old = 0, 56–65 years old = 1, 66–75 years old =2, 75 years old and above =3
Edu	Education level	Illiterate = 0, elementary = 1, middle school and above = 2
Marriage	Marital Status	Divorced or widowed = 0, married = 1. Single = 2
Residence	Nature of permanent residence	Rural residence = 0, urban residence = 1
Fall	Has fallen in the last two years	No = 0, Yes = 1
ADL	Long-term living capacity impairment	No = 0, Yes = 1
lgPCE	Annual household expenditure *per capita*	Logarithm, RMB

Regarding the selection of control variables, we particularly emphasize that this study measures expenditure rather than income as an economic status variable. This is due to the high degree of measurement error in income and the significant variation of income over the life cycle, especially in rural areas where income is affected by factors such as natural disasters. In contrast, expenditure changes less over time, as each household tries to average their consumption across periods (47).

Table 3 presents the descriptive statistics of the variables included in the regression model. The mean score of the dependent variable, depression level score, was 8.34, indicating the majority of the sample population was in a non-depressed state, based on the cut-off point of 10 as suggested by Andresen et al. (48). Among the main variables, the number of caregivers ranged from 0 to 5, with a mean value of 0.4, suggesting that most middle-aged and older adults did not provide intergenerational care or only cared for one grandchild. Among the control variables, the mean age of middle-aged and older adults in the sample was 61.63, and this variable was segmented in the regression model after age stratification, with a mean value of 1.13, corresponding to the age range of 55–65. The mean value of the education level variable was 0.42, indicating that most middle-aged and older adults people are illiterate or have primary education. The mean value of the marital status variable was 1.19, suggesting that the proportion of married middle-aged and older adults people was high. The *per capita* expenditure of middle-aged and older adults individual households with permanent population is 9,206 yuan per person, reflecting the economic status of the sample population.

**Table 3:** Descriptive statistics.

Variable	Mean	SD	Min	Max	Obs.
Dependent variable					
Depression score	8.340	5.950	0	30	6,602
Key Variables					
Whether to provide care	0.520	0.500	0	1	6,602
Number of grandchildren in care	0.400	0.650	0	5	6,602
Length of care	22.71	30.78	0	260	6,596
Control variables					
Age	61.63	8.940	45	98	6,602
Age (segment)	1.130	0.890	0	3	6,602
Education level	0.420	0.690	0	2	6,601
Marital Status	1.190	0.390	0	2	6,602
Nature of permanent residence	0.410	0.490	0	1	6,058
Annual household expenditure *per capita*	9,206	16,740	40	360,000	6,460
Annual household expenditure *per capita* (take logarithm)	8.580	1.020	3.690	12.80	6,460
Falls	0.170	0.380	0	1	6,600
Ability to perform activities of daily living	0.280	0.450	0	1	6,602

## 4. Main results

In the empirical analysis, we began by investigating the impact of intergenerational caregiving on the psychological health of middle-aged and older adults using the proposed model. Subsequently, we explored various intergenerational caregiving variables that captured the intensity of intergenerational caregiving, considering factors such as the amount and duration of caregiving provided. Robustness tests were then conducted to ensure the reliability and consistency of the model. Finally, we examined the differential effects of sample heterogeneity on the psychological health of middle-aged and older adults, taking into account various demographic and socioeconomic characteristics.

### 4.1. Static results

First, we introduced a dummy variable to examine the direct effect of providing intergenerational care on the mental health of middle-aged and older adults. In column (1) of Table 4, only the effect of intergenerational care provision on the mental health of middle-aged and older adults was considered, and the results showed that middle-aged and older adults who provided intergenerational care had significantly lower depression scales and better mental health than those who did not provide such care. Subsequently, demographic variables such as gender, age, education level, marital status, and nature of usual residence, were added one by one in columns (2)–(6). The model’s explanatory power improved as the coefficients of determination increased. The significance of providing intergenerational care on mental health persisted even after incorporating the annual *per capita* household expenditure variable in column (7) and the recent and long-term physical condition variables of the older adults in columns (8) and (9). The final regression results are shown in column (9) of Table 2, where it is evident that providing intergenerational care has a positive effect on the mental health of middle-aged and older adults. Specifically, compared to those who do not provide intergenerational care, individuals who provide such care experience a decrease in their depression scores by approximately 0.4. This finding supports the perspectives of Jing et al. (21) and Coall and Hertwig (20). Possible reasons for this finding can be attributed to the influences of traditional family values and the realization of self-efficacy among middle-aged and older adults individuals in China, as suggested by Jing et al. (21).

**Table 4:** Effects of intergenerational caregiving on the mental health of middle-aged and older adults.

	(1)	(2)	(3)	(4)	(5)	(6)	(7)	(8)	(9)
Provision of intergenerational care (not available = 0)	−0.4612***	−0.5537***	−0.5400***	−0.4682***	−0.4307***	−0.3772**	−0.3670**	−0.3677**	−0.4025***
	(−3.1500)	(−3.8397)	(−3.6280)	(−3.1656)	(−2.9146)	(−2.4615)	(−2.3664)	(−2.3970)	(−2.7294)
Male (Female = 0)		−2.1256***	−2.2047***	−1.9088***	−1.7362***	−1.7856***	−1.7778***	−1.6842***	−1.4494***
		(−14.7491)	(−15.1305)	(−12.8945)	(−11.4602)	(−11.3734)	(−11.1795)	(−10.6912)	(−9.5469)
56–65 years old (Not = 0)			0.5473***	0.2691	0.1785	0.2274	0.1937	0.1264	−0.0643
			(3.0345)	(1.4786)	(0.9781)	(1.2022)	(1.0140)	(0.6687)	(−0.3536)
66–75 years old			0.6959***	0.3367	0.1089	0.2296	0.2298	0.0732	−0.3788*
			(3.3182)	(1.5867)	(0.5035)	(1.0208)	(1.0089)	(0.3243)	(−1.7380)
75+ years old			0.1068	−0.3944	−0.8557***	−0.7498**	−0.8430**	−1.0180***	−1.8753***
			(0.3530)	(−1.2880)	(−2.6857)	(−2.2816)	(−2.4994)	(−3.0481)	(−5.7981)
Elementary school (illiteracy = 0)				−1.0513***	−1.0166***	−0.9479***	−0.9346***	−0.9627***	−0.6280***
				(−5.5027)	(−5.3273)	(−4.7781)	(−4.6688)	(−4.8616)	(−3.2883)
Junior high school and above				−2.2303***	−2.1838***	−2.1176***	−2.0389***	−2.0386***	−1.6712***
				(−9.6321)	(−9.4418)	(−8.6486)	(−8.1949)	(−8.2839)	(−7.0461)
Married (divorced or widowed = 0)					−1.0262***	−0.8914***	−0.9744***	−0.9455***	−0.7483***
					(−5.1741)	(−4.3404)	(−4.6722)	(−4.5827)	(−3.7685)
Unmarried					0.7026	0.6060	0.6715	0.7167	0.4218
					(0.3419)	(0.2970)	(0.3083)	(0.3326)	(0.2036)
Urban (Rural = 0)						−0.6317***	−0.6207***	−0.6263***	−0.3650**
						(−4.1635)	(−4.0476)	(−4.1286)	(−2.4948)
Annual household expenditure *per capita* (Take logarithm)							−0.1067	−0.1282*	−0.1281*
							(−1.4262)	(−1.7312)	(−1.7988)
Fall (never fall = 0)								2.2612***	1.6549***
								(11.4360)	(8.6160)
Impaired ability to perform daily living (no impairment = 0)									3.6499***
									(22.0364)
Constant	8.5757***	9.6815***	9.3074***	9.8276***	10.6704***	10.7074***	11.6811***	11.4991***	10.3797***
	(81.4682)	(75.7165)	(52.5495)	(53.0303)	(43.1934)	(41.2539)	(16.7486)	(16.6636)	(15.6000)
*r*2	0.002	0.033	0.036	0.051	0.055	0.057	0.058	0.079	0.148
*N*	6,602	6,602	6,602	6,601	6,601	6,057	5,927	5,926	5,926

Note: Robust standard errors are in parentheses; ***p < 0.01, **p < 0.05, *p < 0.1.

Among the control variables, gender, age, education, nature of permanent residence, and annual *per capita* household expenditure exhibited significant negative effects on depression among middle-aged and older adults. In comparison to women, men had significantly lower depression scores, with a difference of approximately 1.4 points. Furthermore, compared to individuals aged 45–55 years, depression scores were significantly lower by 0.06 points for those aged 56–65 years, 0.37 points for those aged 66–75 years and above, and 1.8 points for those aged 75 years and above. Higher education level were associated with lower depression scores, with individuals with primary education displaying a decrease of about 0.6 points compared to those who were illiterate. Additionally, individuals with primary and junior high school education had significantly lower depression scores by 0.6 points and 1.7 points, respectively, compared to illiterate individuals. Finally, urban middle-aged and older adults had significantly lower depression scores by 0.36 points compared to their rural counterparts.

On the other hand, the number of falls, a recent physical condition variable measuring physical health, and the impairment in daily living, a long-term physical condition variable, had a significantly positive effect on depression among middle-aged and older adults. Middle-aged and older adults with a higher frequency of falls had significantly increased depression scores by 1.6 points compared to those with better recent physical status. Similarly, individuals with greater impairment in daily living experienced a significant increase in depression scores by 3.6 points compared to those with better long-term physical status.

### 4.2. Robustness

To examine the robustness of the effect of intergenerational caregiving on the mental health of middle-aged and older adults, this study investigated the intensity of intergenerational caregiving. Specifically, the intergenerational caregiving intensity was measured in terms of the number of grandchildren cared for by middle-aged older adults and the number of weeks of caregiving for grandchildren. The regression results presented in Table 5 demonstrated that the effect of intergenerational caregiving on the psychological well-being of middle-aged and older adults remains significantly positive regardless of the construct.

**Table 5:** Different ways of constructing intergenerational care variables.

	CESDscore	CESDscore	CESDscore	CESDscore
Number of grandchildren in care	−0.273**	−0.370***		
	(0.11)	(0.11)		
Weeks of caring for grandchildren			−0.00401*	−0.00648***
			(0.00)	(0.00)
Male (Female = 0)		−1.453***		−1.470***
		(0.15)		(0.15)
56–65 years old (Not = 0)		−0.0471		−0.0387
		(0.18)		(0.18)
66–75 years old		−0.373*		−0.343
		(0.22)		(0.22)
75+ years old		−1.842***		−1.820***
		(0.32)		(0.32)
Elementary school (illiteracy = 0)		−0.626***		−0.632***
		(0.19)		(0.19)
Junior high school and above		−1.669***		−1.684***
		(0.24)		(0.24)
Married (divorced or widowed = 0)		−0.769***		−0.751***
		(0.20)		(0.20)
Unmarried		0.457		0.497
		(2.07)		(2.07)
Urban (Rural = 0)		−0.366**		−0.369**
		(0.15)		(0.15)
Annual household expenditure *per capita* (Take logarithm)		−0.127*		−0.134*
		(0.07)		(0.07)
Fall (never fall = 0)		1.652***		1.651***
		(0.19)		(0.19)
Impaired ability to perform daily living (no impairment = 0)		3.653***		3.662***
(无障碍 = 0)		(0.17)		(0.17)
_cons	8.445***	10.78***	8.431***	10.86***
	(0.09)	(2.15)	(0.09)	(2.15)
N	6,602	5,926	6,600	5,924
r2	0.000889	0.149	0.000489	0.149

First, the regression analysis considers the number of grandchildren cared for. In real-life scenarios, middle-aged or older adults individuals may provide care for multiple grandchildren simultaneously. Within the sample analyzed in this study, it was observed that some older adults individuals cared for up to five grandchildren. The regression results, as shown in columns (1) and (2), closely align with the findings obtained from the dummy variables used in the baseline regression.

Second, the regression analysis incorporates the duration of caregiving in weeks. Due to intra-household dynamics, the number of hours dedicated to caregiving for grandchildren varies among older adults individuals. In constructing the measure of caregiving duration, this study adopts an approach of summing the number of weeks spent by older adults in providing care to their grandchildren. This approach accounts for the fact that the intensity of caregiving for one grandchild over a span of 10 weeks differs from that of caregiving for two grandchildren over the same duration. With the sample, the older adults individuals who provided care for the longest duration accumulated up to 260 weeks. The regression results, as shown in columns (3) and (4), exhibit consistent directions of impact with the dummy variables used in the baseline regression.

### 4.3. Heterogeneity

We have established that providing intergenerational care has a significant positive effect on the mental health of middle-aged and older adults. In this section, we explore whether this effect varies across different groups of older adults.

1. Gender: When examining the gender subsample of middle-aged and older adults, it was found that providing care for grandchildren positively influenced their mental health for both males and females. However, the positive effect was more pronounced for males compared to females. It could be attributed to several factors. Firstly, cultural norms and expectations regarding gender roles may play a role. In many societies, including certain regions of China, men are traditionally considered the primary breadwinners and may have fewer caregiving responsibilities compared to women. As a result, when men engage in grandparent caregiving, it may be seen as a more novel and fulfilling experience, leading to a greater sense of purpose and satisfaction, thus positively impacting their mental well-being. Secondly, the gender differences could be influenced by the dynamics of intergenerational relationships. Research suggests that the relationship between grandfathers and grandchildren can be particularly special and unique. Grandfathers may engage in activities with their grandchildren that are perceived as more recreational or leisure-oriented, such as playing sports or engaging in hobbies together. These interactions may contribute to increased emotional well-being and a sense of joy and rejuvenation, thus positively affecting the mental health of grandfathers.
2. Social resources: Categorizing middle-aged and older adults by age group, intergenerational care significantly improved the mental health of those aged 45–55 and 56–65. However, the effects were not significant for middle-aged and older adults aged 65–75 and those older than 75. Also, based on the permanent residence of middle-aged older adults, providing intergenerational care had a significant positive effect on urban individuals and a smaller, insignificant positive effect on rural individuals. And when considering the educational level of the middle-aged and older adults, the positive effect of intergenerational care mental health was higher for those with a higher educational level. This effect was particularly significant for those with limited education, such as those who were illiterate or had primary education. The above results may be attributed to social resources, as disadvantaged middle-aged and older groups often have limited access to socioeconomic and caregiving resources. Providing intergenerational care increases their physical and mental stress to a greater extent, with fewer alternative avenues for stress relief. For example, individuals with higher education may have more hobbies and participate in more community activities to alleviate stress, whereas older adults, as found by Li et al. (49), are less likely to engage in such activities. Moreover, urban seniors often have access to senior centers and in-home senior care services, while rural seniors face challenges in accessing these resources, which may contribute to higher stress levels associated with providing intergenerational care.
3. Family structure: Intergenerational care had a significant positive effect on the mental health of married middle-aged and older adults. However, it had little or no effect on divorced and widowed middle-aged and older adults. Family structure and internal relationships also play a role. In families with living grandparents, grandmothers typically shoulder more caregiving responsibilities due to traditional gender roles (50). In divorced or widowed families, the grandfather or grandmother may be the sole caregiver, leading to higher stress levels compared to married families where caregiving responsibilities are shared. These factors may contribute to a smaller positive impact intergenerational care in these circumstances.
4. Physical condition: Categorizing individuals based on their physical health, the provision of intergenerational care had a stronger positive and significant effect on the mental health of both the middle-aged and older adults with good recent physical health and good long-term physical health. The physical condition of the caregiver influences the magnitude of positive effect. The caregiver with poorer health increases both physical and emotional stress associated with providing care and may result in less frequent or delayed access to medical care, thereby diminishing the positive effect of intergenerational care.

By considering these factors, we gain insights into the varying effects of intergenerational caregiving on the mental health of different subgroups of middle-aged and older adults. These findings shed light on the complex interplay between intergenerational caregiving, sociodemographic factors, family dynamics, and health status (Table 6).

**Table 6:** Heterogeneity.

CESD_score				
Sample by gender				
Provide intergenerational care (not available = 0)	Female	Male		
	−0.357	−0.477**		
	(0.23)	(0.19)		
*N*	2,968	2,958		
Sample by nature of permanent residence				
Provide intergenerational care (not available = 0)	Rural	Urban		
	−0.196	−0.648***		
*N*	(0.19)	(0.23)		
Sample by age				
Provide intergenerational care (not available = 0)	45–55	56–65	66–75	75+
	−0.670**	−0.519**	−0.0682	0.0953
	(0.29)	(0.22)	(0.30)	(0.67)
*N*	1,552	2,558	1,369	447
Sample by education level				
Provide intergenerational care (not available = 0)	Illiterate	Primary school		Junior high school and above
	−0.333*	−0.622*		−0.806
	(0.18)	(0.32)		(0.38)
*N*	4,121	1,143		662
Sample by marital status				
Provide intergenerational care (not available = 0)	Married	Divorced or widowed		
	−0.472***	−0.0375		
	(0.16)	(0.39)		
*N*	4,836	1,083		
Sample by whether you have fallen (recent physical condition)				
Provide intergenerational care (not available = 0)	No	Yes		
	−0.443***	−0.227		
	(0.16)	(0.39)		
*N*	4,928	998		
Sample by ability to live on their own (long-term physical condition)				
Provide intergenerational care (not available = 0)	No	Yes		
	−0.488***	−0.215		
	(0.16)	(0.34)		
*N*	4,303	1,623		

## 5. Mechanism

Following our examination of the facilitative effect of intergenerational caregiving on the mental health of middle-aged and older adults, we aimed to explore the underlying mechanisms that drive this effect. Specifically, we investigated how providing care to grandchildren by middle-aged and older adults stimulates certain behaviors and forms of support from the parents of the grandchildren. Our findings indicate that when middle-aged and older adults provide care for their grandchildren, the parents of their grandchildren often reciprocate by increasing their intergenerational support for their own parents. This support can manifest in various forms, including financial assistance, spiritual guidance, and overall life care. In this paper, we focus on intergenerational financial support and intergenerational spiritual support, as life care support was not considered due to factors such as geographical separation between generations or the busy schedules of the younger parents. Intergenerational support has two long-term effects on the mental health status of middle-aged and older parents. Firstly, when children provide intergenerational economic support, they contribute more financial resources to their parents, which improves the economic well-being of middle-aged and older adults. This enhanced financial situation allows them to experience greater satisfaction, leading to an improved quality of life, increased life satisfaction, and ultimately better mental health. Secondly, intergenerational spiritual support, such as intergenerational care provided by middle-aged and older adults to their grandchildren, fosters increased communication channels between generations. This facilitates the release of stress, provides spiritual comfort, and enhances the overall sense of well-being for middle-aged and older adults, thereby improving their psychological health. Therefore, we propose that the promotion of intergenerational caregiving on the psychological health of middle-aged and older adults may operate through two distinct channels: intergenerational economic support and intergenerational spiritual support. The provision of financial assistance and the nurturing of intergenerational relationships contribute to the positive impact on mental health observed in this study.

### 5.1. Intergenerational economic support

To explore the potential mediating role of financial support, this study employed a measure of financial support intensity based on the total amount of money provided by children who entrusted intergenerational care to their parents in the past year. The CHARLS questionnaire included the question, “In the past year, how much financial support did you or your spouse receive from a child who does not have the name of the child you live with?” In this paper, the variable “ trans_money ” was constructed to represent intergenerational financial support by taking the logarithm of the amount.


$${\mathrm{trans}}_{\mathrm{money}}=\beta_{0}+\beta_{1}\mathrm{babysit}+\mathrm{Control}+\sigma$$

$$\mathrm{CESDscore}=\alpha_{2}+\beta_{2}\mathrm{babysit}+\mathrm{Control}+\sigma$$

(2) $$\mathrm{CESDscore}=\alpha_{3}+\beta_{3}\mathrm{babysit}+\gamma_{3}\mathrm{trans}\_\mathrm{money}+\mathrm{Control}+\sigma$$

We tested the mediating effects through the following model: if intergenerational care exerts a partial effect through the mediating mechanism of intergenerational financial support, two conditions must be satisfied: first, the intergenerational care variable (babysit) should facilitate intergenerational financial support as indicated by the total amount of “ trans_money ” remitted by the child to the parent in the past year ( $\beta_{1}$ > 0); second, in the decision equation of CESD score, the mediating variable “ trans_money ” should have a significant negative effect ( $\gamma_{3}$ < 0), and the coefficient of the intergenerational care variable should decrease after the introduction of the mediating variable ( $\beta_{2}>\beta_{3}$ ; Table 7).

**Table 7:** Intergenerational channels of economic support.

	(1)	(2)	(3)
	Intergenerational economic support	CESDscore	CESDscore
Provide intergenerational care (not available = 0)	0.265***	−0.403***	−0.148
	(0.04)	(0.15)	(0.17)
Control variables	Y	Y	Y
Intergenerational economic support			−0.367***
			(0.06)
Constant	5.206***	10.80***	11.43***
	(0.62)	(2.15)	(2.56)
N	4,378	5,926	4,378
r2	0.0757	0.148	0.156

The empirical findings demonstrate that providing intergenerational care significantly increases the number of past-year remittances and overall intergenerational economic support from children. Moreover, the coefficient of the intergenerational care variable changes from-0.403 to-0.148 after the introduction of the intergenerational economic support variable in column (3), indicating the existence of intergenerational economic support as a mediating mechanism in the positive effect of intergenerational care on the mental health of middle-aged and older adults. This suggests that intergenerational financial support partially mediates the relationship between intergenerational care and mental health outcomes.

### 5.2. Intergenerational spiritual support

In examining the mediating mechanism of intergenerational spiritual support, this study assessed children’s intergenerational spiritual support for their parents through the frequency of their interactions. The household questionnaire in the CHARLS data provided the response options for the question “How often do you see this daughter or son?” as follows: 1. Almost every day, 2. 2–3 times a week, 3. 1 time a week, 4. 1 time every half month, 5. 1 time a month, 6. 1 time every 3 months, 7. once every 6 months, 8. once a year, 9. almost never. Based on the frequency of interactions, responses 5–9 were considered low intensity and assigned a value of 0, responses 2–4 were considered medium intensity and assigned a value of 1, and response 1 was considered high intensity and assigned a value of 2.

The empirical findings reveal that in both (1) ordered logit model and (4) ordered probit model, the coefficient of the intergenerational care variable is significantly positive, indicating that providing intergenerational care significantly increases children’s intergenerational spiritual support. Furthermore, after the introduction of the child intergenerational spiritual support variable in column (3), parents who received moderate and high intensity intergenerational spiritual support to their children experienced a significant reduction of about 0.7 points in their depression scale scores compared to those who received low intensity intergenerational spiritual support. At the same time, the coefficient of the intergenerational care variable increased from-0.4 to-0.34, indicating that the positive effect of intergenerational care on the mental health of middle-aged and older adults was mediated by the intergenerational spiritual support. Thus, intergenerational spiritual support serves as a mediating mechanism in this context (Table 8).

**Table 8:** Intergenerational channels of spiritual support.

	(1) Ordered logit model	(2)	(3)	(4) Ordered logit model	(5)	(6)
	Intergenerational spiritual support	CESDsocre	CESDsocre	Intergenerational spiritual support	CESDsocre	CESDsocre
Provide intergenerational care (not available = 0)	0.358***	−0.403***	−0.346**	0.204***	−0.403***	−0.346**
	(0.06)	(0.15)	(0.15)	(0.03)	(0.15)	(0.15)
Control	Y	Y	Y	Y	Y	Y
Medium-intensity intergenerational spiritual support (Low intensity = 0)			−0.732***			−0.732***
			(0.25)			(0.25)
High intensity intergenerational spiritual support			−0.723***			−0.723***
			(0.19)			(0.19)
_cons		10.80***	11.30***		10.80***	11.30***
		(2.15)	(2.15)		(2.15)	(2.15)
cut1	−1.845**			−1.033**		
	(0.76)			(0.49)		
cut2	−1.075			−0.580		
	(0.76)			(0.49)		
*N*	5,845	5,926	5,845	5,845	5,926	5,845
*r*2		0.148	0.151		0.148	0.151

## 6. Conclusion

As mental health issues continue to be a growing concern among the older adults, this paper aims to investigate the factors and mechanisms affecting the mental health of middle-aged and older adults individuals engaged in intergenerational caregiving, using the current caregiving situation in China as a basis. The empirical results support the views of scholars such as Sun (36) and Huang Guogui (51) that intergenerational caregiving has a significant positive impact on the mental health of middle-aged and older adults individuals. The sample heterogeneity analysis concluded that while intergenerational caregiving is prevalent in China, different population groups may face varying contexts and pressures. For instance, older adults individuals in rural areas may experience greater pressure and responsibility in intergenerational caregiving, as they are often responsible for taking care of their grandchildren. Additionally, older adults with lower levels of education may encounter specific challenges in intergenerational caregiving, such as uncertainty and anxiety regarding the education of their grandchildren.

Furthermore, we conducted a robustness check on the intensity of intergenerational care by exploring the number and the length of intergenerational caregivers, as well as the way the intergenerational caregiving variable is constructed. The results confirmed the robustness of our findings. In addition, we investigated the effect of intergenerational caregiving on the mental health of middle-aged and older adults through two mediating mechanisms: intergenerational financial support and intergenerational spiritual support from children. The findings suggest that young children should increase their spiritual care and financial support to their middle-aged and older adults parents.

By studying the impact of intergenerational caregiving on mental health, we can propose more accurate policies and intervention measures. The government can increase support for rural areas, the older adults, and older adults with lower levels of education by providing more social care and economic assistance. Additionally, the government can strengthen support for women, encouraging their active participation in intergenerational caregiving and providing appropriate resources and support. Moreover, improving healthcare utilization among older adults with poor health is also an important goal. This can be achieved by providing easier access to healthcare services and health education to promote their mental well-being.

Further research and exploration in this field are still needed. By delving deeper into studying the mechanisms through which intergenerational caregiving influences mental health, as well as its effects on specific population groups and contexts, including potential negative consequences, we can provide policymakers with more comprehensive recommendations. These recommendations can contribute to improving the mental well-being of middle-aged and older individuals and promoting the sustainable development of intergenerational caregiving.

## Data availability statement

The original contributions presented in the study are included in the article/supplementary material, further inquiries can be directed to the corresponding authors.

## Author contributions

SY, XY, and SL conceived, designed, performed, and analyzed the research. SY and XY conceived, designed, and collected data from website. SY carried out the statistical analyzes. XY and SL wrote the manuscript. All authors read, review and approved the final manuscript.

## Funding

This study was supported by Ministry of Education, Humanities and Social Sciences Youth Fund (21YJC790138), National Natural Science Youth Fund (72103151), and Tianjin Science and Technology Project (18ZXRHSY00100).

## Conflict of interest

The authors declare that the research was conducted in the absence of any commercial or financial relationships that could be construed as a potential conflict of interest.

## Publisher’s note

All claims expressed in this article are solely those of the authors and do not necessarily represent those of their affiliated organizations, or those of the publisher, the editors and the reviewers. Any product that may be evaluated in this article, or claim that may be made by its manufacturer, is not guaranteed or endorsed by the publisher.
